# Empagliflozin reduces cardiovascular events, mortality and renal events in participants with type 2 diabetes after coronary artery bypass graft surgery: subanalysis of the EMPA-REG OUTCOME® randomised trial

**DOI:** 10.1007/s00125-018-4644-9

**Published:** 2018-05-19

**Authors:** Subodh Verma, C. David Mazer, David Fitchett, Silvio E. Inzucchi, Egon Pfarr, Jyothis T. George, Bernard Zinman

**Affiliations:** 1 Division of Cardiac Surgery, St Michael’s Hospital, University of Toronto, 30 Bond St, Toronto, ON M5B 1W8 Canada; 2 Department of Anesthesia, St Michael’s Hospital, University of Toronto, Toronto, ON Canada; 3 Division of Cardiology, St Michael’s Hospital, University of Toronto, Toronto, ON Canada; 4 Section of Endocrinology, Yale University School of Medicine, New Haven, CT USA; 5 Boehringer Ingelheim Pharma GmbH & Co. KG, Ingelheim, Germany; 6 Boehringer Ingelheim International GmbH, Ingelheim, Germany; 7 Lunenfeld-Tanenbaum Research Institute, Mount Sinai Hospital, University of Toronto, Toronto, Canada

**Keywords:** Cardiovascular disease, Coronary artery bypass graft, Coronary revascularisation, Diabetes mellitus, Empagliflozin, Sodium glucose cotransporter 2 inhibition, Type 2 diabetes

## Abstract

**Aims/hypothesis:**

After coronary artery bypass graft (CABG) surgery in individuals with type 2 diabetes, there remains a considerable residual cardiovascular risk. In the EMPA-REG OUTCOME® trial in participants with type 2 diabetes and established cardiovascular disease, empagliflozin reduced the risk of cardiovascular death by 38%, all-cause mortality by 32%, hospitalisation for heart failure by 35% and incident or worsening nephropathy by 39% vs placebo when given in addition to standard of care. The aim of this post hoc analysis of the EMPA-REG OUTCOME® trial was to determine the effects of the sodium glucose cotransporter 2 inhibitor empagliflozin on cardiovascular events and mortality in participants with type 2 diabetes and a self-reported history of CABG surgery.

**Methods:**

The EMPA-REG OUTCOME® trial was a randomised, double-blind, placebo-controlled trial. Participants with type 2 diabetes and established cardiovascular disease were randomised 1:1:1 to receive placebo, empagliflozin 10 mg or empagliflozin 25 mg, once daily, in addition to standard of care. In subgroups by self-reported history of CABG (yes/no) at baseline, we assessed: cardiovascular death; all-cause mortality; hospitalisation for heart failure; and incident or worsening nephropathy (progression to macroalbuminuria, doubling of serum creatinine, initiation of renal replacement therapy or death due to renal disease). Differences in risk between empagliflozin and placebo were assessed using a Cox proportional hazards model.

**Results:**

At baseline, 25% (1175/4687) of participants who received empagliflozin and 24% (563/2333) of participants who received placebo had a history of CABG surgery. In participants with a history of CABG surgery, HRs (95% CI) with empagliflozin vs placebo were 0.52 (0.32, 0.84) for cardiovascular mortality, 0.57 (0.39, 0.83) for all-cause mortality, 0.50 (0.32, 0.77) for hospitalisation for heart failure and 0.65 (0.50, 0.84) for incident or worsening nephropathy. Results were consistent between participants with and without a history of CABG surgery (*p* > 0.05 for treatment by subgroup interactions).

**Conclusions/interpretation:**

In participants with type 2 diabetes and a self-reported history of CABG surgery, treatment with empagliflozin was associated with profound reductions in cardiovascular and all-cause mortality, hospitalisation for heart failure, and incident or worsening nephropathy. These data have important implications for the secondary prevention of cardiovascular events after CABG in individuals with type 2 diabetes.

**Trial registration:**

ClinicalTrials.gov NCT01131676

**Electronic supplementary material:**

The online version of this article (10.1007/s00125-018-4644-9) contains peer-reviewed but unedited supplementary material, which is available to authorised users.

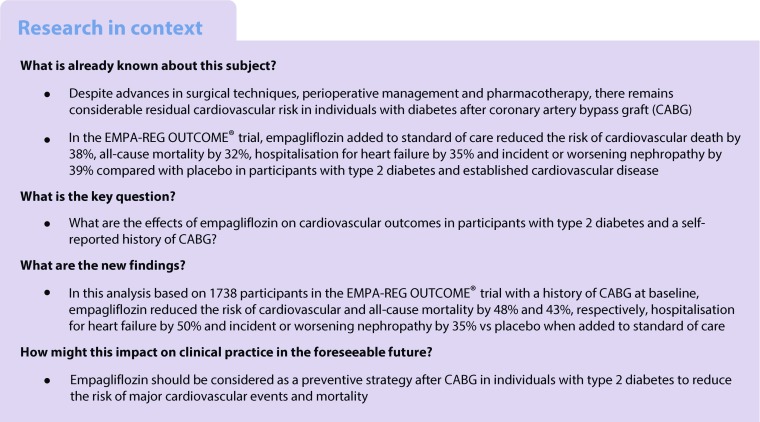



## Introduction

Compared with individuals without diabetes, individuals with diabetes have a higher rate, extent and severity of obstructive coronary artery disease [1]. Individuals with diabetes and multi-vessel coronary artery disease derive greater benefit from coronary artery bypass graft (CABG) surgery than percutaneous coronary intervention (PCI) [2], and CABG is regarded as a preferred strategy in these individuals [3]. However, despite advances in surgical techniques, perioperative management and pharmacotherapy, there remains considerable residual cardiovascular risk in individuals with diabetes after CABG. The 5 year event rate of major adverse cardiovascular events (MACE) after CABG in participants with diabetes was 19% in the FREEDOM trial and 29% in the SYNTAX trial [4, 5]. In addition to residual ischaemic cardiovascular risk, there remains a substantial risk of heart failure after CABG, with 2 year rates of heart failure hospitalisation ranging from 12.9% (in participants with ejection fraction ≥50%) to 36.9% (in those with ejection fraction <25%) [6].

Following Food and Drug Administration (FDA) guidance [7], results are available from large trials evaluating the cardiovascular effects of glucose-lowering agents in participants with type 2 diabetes. One such trial was the EMPA-REG OUTCOME® trial (ClinicalTrials.gov NCT01131676), in which participants with type 2 diabetes and established cardiovascular disease were randomised to receive the sodium glucose cotransporter 2 (SGLT2) inhibitor empagliflozin or placebo in addition to standard of care [8]. The EMPA-REG OUTCOME® trial was the first placebo-controlled trial to report a benefit of a glucose-lowering agent on major cardiovascular outcomes, with risk reductions in cardiovascular and all-cause mortality of 38% and 32%, respectively [8]. Data from this trial led to the FDA approval of an indication for empagliflozin to include reducing the risk of cardiovascular death in participants with type 2 diabetes and established cardiovascular disease—the first such approval for a glucose-lowering agent [9]. These data have also led to recommendations in clinical practice guidelines that empagliflozin should be considered in the treatment of individuals with type 2 diabetes and cardiovascular disease [10, 11].

There remains controversy regarding pharmacological cardiovascular risk reduction approaches in individuals following surgical revascularisation, with very limited data on this group available from large randomised clinical trials [12, 13]. Approximately one quarter of the 7020 participants enrolled in the EMPA-REG OUTCOME® trial had a self-reported history of CABG at baseline. We investigated the effect of empagliflozin on cardiovascular outcomes and mortality in this subgroup.

## Methods

### Trial design and population

The design of the EMPA-REG OUTCOME® trial has been described and the study protocol has been published [8]. Briefly, eligible participants had type 2 diabetes (with HbA_1c_ 53–75 mmol/mol [7.0–9.0%] for drug-naive participants and 53–86 mmol/mol [7.0–10.0%] for those on stable glucose-lowering therapy), established cardiovascular disease and estimated (e)GFR, according to modification of diet in renal disease (MDRD) ≥30 ml min^−1^ 1.73 m^−2^. Participants were identified as having a history of CABG at baseline based on case report forms in which the investigator noted a history of CABG (based on the participant’s self-report) using a yes/no checkbox. Participants were randomised to receive empagliflozin 10 mg, empagliflozin 25 mg or placebo, once daily. Background glucose-lowering therapy was to remain unchanged for 12 weeks. After week 12, investigators were encouraged to adjust glucose-lowering therapy to achieve glycaemic control according to local guidelines. Investigators were encouraged to treat cardiovascular risk factors to achieve the best standard of care according to local guidelines throughout the trial. Serum creatinine and urinary albumin were measured from spot urine samples obtained during study visits at screening (creatinine only), during the placebo run-in period, at baseline, at weeks 12, 28 and 52 and then every 14 weeks until the final visit. Events that were consistent with changes in the albuminuria category were captured if any laboratory assessment during the trial fulfilled the given criteria on at least one occasion. The trial was to continue until ≥691 participants experienced an adjudicated event included in the primary outcome (three-point MACE: composite of cardiovascular death, non-fatal myocardial infarction and non-fatal stroke). Participants who prematurely discontinued study medication continued to be followed for ascertainment of cardiovascular outcomes, adverse events and vital status.

The EMPA-REG OUTCOME® trial was registered with ClinicalTrials.gov (NCT01131676) and carried out in compliance with the protocol and the principles of the Declaration of Helsinki, and in accordance with the International Conference on Harmonization Harmonized Tripartite Guideline for Good Clinical Practice. All participants provided signed and dated informed consent. The trial was conducted at 590 sites in 42 countries.

### Outcomes

All cardiovascular outcome events and deaths were prospectively adjudicated by Clinical Events Committees who were blinded to treatment allocation. The following outcomes were analysed in subgroups by history of CABG (yes/no) at baseline: three-point MACE (composite of cardiovascular death, non-fatal myocardial infarction or non-fatal stroke); fatal or non-fatal myocardial infarction; fatal or non-fatal stroke; cardiovascular death; four-point MACE (composite of cardiovascular death, non-fatal myocardial infarction, non-fatal stroke or hospitalisation for unstable angina); hospitalisation for heart failure; composite of heart failure hospitalisation or cardiovascular death; all-cause mortality; incident or worsening nephropathy (defined as progression to macroalbuminuria [urine albumin-to-creatinine ratio >300 mg/g], doubling of serum creatinine accompanied by eGFR [MDRD] ≤45 ml min^−1^ 1.73 m^−2^, initiation of renal replacement therapy or death due to renal disease). Safety was assessed based on adverse events that occurred during treatment or within 7 days after the last dose of study drug.

### Analyses

Analyses in subgroups by CABG at baseline were post hoc and based on participants who received ≥1 dose of study drug, except for incident or worsening nephropathy. This outcome was analysed in participants who received ≥1 dose of study drug who did not have macroalbuminuria at baseline, who had serum creatinine measurements at baseline and after baseline, and who had post-baseline urine albumin-to-creatinine ratio measurements (unless participants who did not fulfil these criteria had at least one of the other components of the composite renal outcome).

Differences between empagliflozin (pooled) and placebo in the risk of an outcome were assessed using a Cox proportional hazards model, with factors for age, sex, baseline BMI, baseline HbA_1c_, baseline eGFR, region, treatment, CABG and treatment by CABG interaction, using events observed from randomisation to the end of the trial. Adjustment for these factors is consistent with the pre-specified analyses in subgroups by other baseline characteristics [8].

The *p* values for treatment by subgroup interaction were obtained from tests of homogeneity of treatment group differences among subgroups, with no adjustment for multiple testing. Data from participants who did not have an event were censored on the last day they were known to be free of the outcome. Kaplan–Meier estimates are presented for three-point MACE, cardiovascular death, hospitalisation for heart failure, all-cause mortality and incident or worsening nephropathy.

In participants with a history of CABG, we assessed cardiovascular death, hospitalisation for heart failure and all-cause mortality in subgroups by time from CABG to randomisation (≤5, >5 to ≤10, >10 years) and the proportion of participants with different numbers of coronary revascularisation (redo CABG; redo and/or new PCI) procedures during the trial. Outcomes in subgroups by time from CABG to randomisation and the proportion of participants with different numbers of coronary revascularisation procedures during the trial were assessed descriptively. Adverse events in subgroups by history of CABG were assessed descriptively, consistent with how adverse events in other subgroups were to be analysed according to the statistical analysis plan [8].

## Results

### Baseline characteristics

**Fig. 1:**
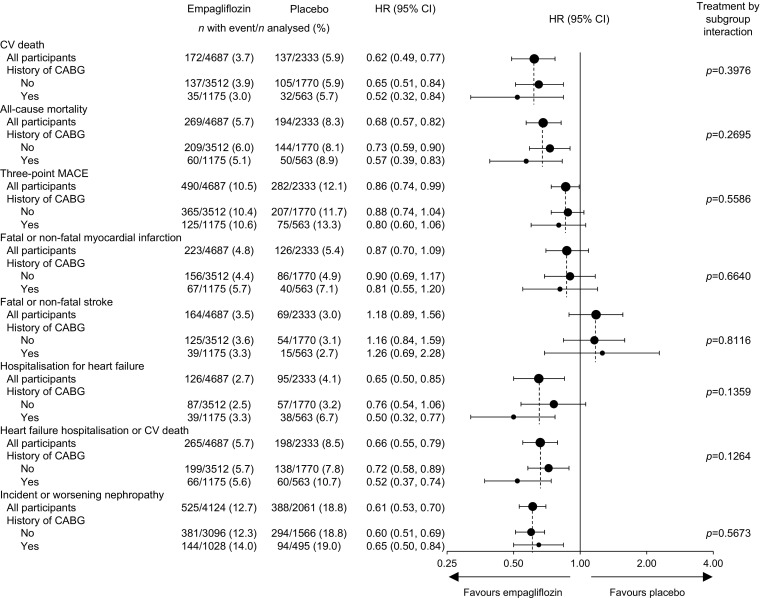
Cardiovascular outcomes, all-cause mortality and incident or worsening nephropathy by history of CABG surgery. Cox regression analysis in participants treated with ≥1 dose of study drug, except for incident or worsening nephropathy, which was analysed in participants who received ≥1 dose of study drug who did not have macroalbuminuria at baseline, had serum creatinine measurements at baseline and after baseline, and had post-baseline urine albumin-to-creatinine ratio measurements. Interaction *p* value is for test of homogeneity of treatment group difference among subgroups (test for treatment by subgroup interaction) with no adjustment for multiple tests. CV; cardiovascular

Between 1 September 2010 and 22 April 2013, 7028 participants were randomised and 7020 participants received the study drug (electronic supplementary material [ESM] Fig. 1). At baseline, a history of CABG was present in 25% (1175/4687) of participants in the empagliflozin (pooled) group and 24% (563/2333) of participants in the placebo group. Baseline characteristics were balanced between the empagliflozin and placebo groups in participants with and without CABG (Table 1). At baseline, mean (SD) age of participants with a history of CABG was 65 (8) years, HbA_1c_ was 64 (9.2) mmol/mol (8.05 [0.84]%), 81% were male, 51% had a history of myocardial infarction, 11% had a history of heart failure, 15%, 52% and 33% had eGFR ≥90, 60 to <90 and <60 ml min^−1^ [1.73 m]^−2^, respectively, mean (SD) systolic blood pressure was 135.6 (17.1) mmHg and diastolic blood pressure was 75.3 (10.0) mmHg. The median observation time was 3.1 years.

**Table 1:** Baseline characteristics by history of CABG at baseline

Characteristic	Participants with a history of CABG		Participants without a history of CABG	
	Empagliflozin (*n* = 1175)	Placebo (*n* = 563)	Empagliflozin (*n* = 3512)	Placebo (*n* = 1770)
Age, years	64.5 ± 8.2	65.5 ± 8.0	62.6 ± 8.6	62.5 ± 8.9
Male	945 (80.4)	459 (81.5)	2391 (68.1)	1221 (69.0)
Race				
White	922 (78.5)	441 (78.3)	2481 (70.6)	1237 (69.9)
Asian	191 (16.3)	78 (13.9)	815 (23.2)	433 (24.5)
Black/African-American	51 (4.3)	35 (6.2)	186 (5.3)	85 (4.8)
Other/Missing	11 (0.9)	9 (1.6)	30 (0.9)	15 (0.9)
Region				
Europe	399 (34.0)	189 (33.6)	1527 (43.5)	770 (43.5)
North America (plus Australia and New Zealand)	389 (33.1)	201 (35.7)	543 (15.5)	261 (14.7)
Asia	138 (11.7)	58 (10.3)	759 (21.6)	392 (22.1)
Latin America	154 (13.1)	72 (12.8)	567 (16.1)	288 (16.3)
Africa	95 (8.1)	43 (7.6)	116 (3.3)	59 (3.3)
Weight, kg	88.8 ± 18.6	90.5 ± 19.7	85.3 ± 18.9	85.4 ± 18.7
BMI, kg/m^2^	30.8 ± 5.1	31.2 ± 5.2	30.5 ± 5.3	30.5 ± 5.2
Time since CABG				
≤1 year	86 (7.3)	33 (5.9)	n/a	n/a
>1 to 5 years	386 (32.9)	174 (30.9)	n/a	n/a
>5 to 10 years	373 (31.7)	170 (30.2)	n/a	n/a
>10 years	322 (27.4)	181 (32.1)	n/a	n/a
CV disease				
Coronary artery disease	1175 (100.0)	563 (100.0)	2370 (67.5)	1200 (67.8)
Multi-vessel coronary artery disease	869 (74.0)	420 (74.6)	1310 (37.3)	680 (38.4)
History of myocardial infarction	598 (50.9)	286 (50.8)	1592 (45.3)	797 (45.0)
History of stroke^a^	140 (11.9)	59 (10.5)	944 (26.9)	494 (27.9)
Peripheral artery disease^b^	216 (18.4)	107 (19.0)	766 (21.8)	372 (21.0)
Single vessel coronary artery disease^a^	60 (5.1)	25 (4.4)	438 (12.5)	213 (12.0)
Cardiac failure^c^	119 (10.1)	70 (12.4)	343 (9.8)	174 (9.8)
HbA_1c_, mmol/mol^d^	64 ± 9.1	64 ± 9.3	65 ± 9.3	65 ± 9.2
HbA_1c_, %^d^	8.05 ± 0.83	8.04 ± 0.85	8.08 ± 0.85	8.09 ± 0.84
Time since diagnosis of type 2 diabetes				
≤1 year	18 (1.5)	7 (1.2)	110 (3.1)	45 (2.5)
>1 to 5 years	125 (10.6)	63 (11.2)	587 (16.7)	308 (17.4)
>5 to 10 years	277 (23.6)	112 (19.9)	898 (25.6)	459 (25.9)
>10 years	755 (64.3)	381 (67.7)	1917 (54.6)	958 (54.1)
Glucose-lowering therapy				
Medication taken alone or in combination				
Metformin	840 (71.5)	411 (73.0)	2619 (74.6)	1323 (74.7)
Sulfonylurea	440 (37.4)	220 (39.1)	1574 (44.8)	772 (43.6)
Insulin	675 (57.4)	318 (56.5)	1577 (44.9)	817 (46.2)
Monotherapy	340 (28.9)	171 (30.4)	1040 (29.6)	520 (29.4)
Dual therapy	570 (48.5)	252 (44.8)	1689 (48.1)	896 (50.6)
Anti-hypertensive therapy	1127 (95.9)	550 (97.7)	3319 (94.5)	1671 (94.4)
Angiotensin-converting enzyme inhibitor/angiotensin receptor blocker	961 (81.8)	452 (80.3)	2837 (80.8)	1416 (80.0)
Beta-blocker	902 (76.8)	434 (77.1)	2154 (61.3)	1064 (60.1)
Diuretic	572 (48.7)	281 (49.9)	1475 (42.0)	707 (39.9)
Calcium channel blocker	359 (30.6)	187 (33.2)	1170 (33.3)	601 (34.0)
Mineralocorticoid receptor antagonist	96 (8.2)	36 (6.4)	209 (6.0)	100 (5.6)
Renin inhibitor	10 (0.9)	5 (0.9)	17 (0.5)	14 (0.8)
Other	88 (7.5)	50 (8.9)	295 (8.4)	141 (8.0)
Lipid-lowering therapy	1025 (87.2)	501 (89.0)	2795 (79.6)	1363 (77.0)
Statin	974 (82.9)	481 (85.4)	2656 (75.6)	1292 (73.0)
Fibrate	116 (9.9)	50 (8.9)	315 (9.0)	149 (8.4)
Ezetimibe	65 (5.5)	26 (4.6)	124 (3.5)	55 (3.1)
Niacin	40 (3.4)	13 (2.3)	51 (1.5)	22 (1.2)
Other	137 (11.7)	69 (12.3)	228 (6.5)	106 (6.0)
Anticoagulant	1098 (93.4)	536 (95.2)	3064 (87.2)	1554 (87.8)
Acetylsalicylic acid	1035 (88.1)	496 (88.1)	2841 (80.9)	1431 (80.8)
Clopidogrel	98 (8.3)	48 (8.5)	396 (11.3)	201 (11.4)
Vitamin K antagonist	79 (6.7)	59 (10.5)	187 (5.3)	97 (5.5)
Systolic blood pressure, mmHg	135.5 ± 17.0	136.1 ± 17.3	135.2 ± 16.9	135.7 ± 17.2
Diastolic blood pressure, mmHg	75.3 ± 9.8	75.2 ± 10.6	77.1 ± 9.7	77.3 ± 10.0
Total cholesterol, mmol/l^e^	4.1 ± 1.0	4.0 ± 1.1	4.3 ± 1.2	4.2 ± 1.1
LDL-cholesterol, mmol/l^f^	2.1 ± 0.9	2.1 ± 0.9	2.3 ± 1.0	2.2 ± 0.9
HDL-cholesterol, mmol/l^e^	1.1 ± 0.3	1.1 ± 0.3	1.2 ± 0.3	1.2 ± 0.3
Triacylglycerol, mmol/l^e^	1.8 ± 1.1	1.9 ± 1.4	2.0 ± 1.6	1.9 ± 1.4
eGFR (MDRD), ml min^−1^ 1.73 m^−2^	69.8 ± 20.2	68.9 ± 20.1	75.6 ± 21.8	75.4 ± 21.1
≥90	183 (15.6)	77 (13.7)	867 (24.7)	411 (23.2)
60 to <90	612 (52.1)	290 (51.5)	1811 (51.6)	948 (53.6)
<60	380 (32.3)	196 (34.8)	832 (23.7)	411 (23.2)

Data are *n* (%) or mean ± SD in participants treated with ≥1 dose of study drug

^a^Information was not available for one participant in the placebo group

^b^Information was not available for one participant in the placebo group and one participant in the empagliflozin group

^c^Based on the narrow standardised Medical Dictionary for Regulatory Activities (MedDRA) query ‘cardiac failure’

^d^Information was not available for one participant in the empagliflozin group

^e^Empagliflozin *n* = 1162 and placebo *n* = 555 for participants with a history of CABG; empagliflozin *n* = 3464 and placebo *n* = 1754 for participants without a history of CABG

^f^Empagliflozin *n* = 1161 and placebo *n* = 555 for participants with a history of CABG; empagliflozin *n* = 3462 and placebo *n* = 1754 for participants without a history of CABG

CV, cardiovascular; n/a, not applicable

Background medication use was balanced between the empagliflozin and placebo groups in participants with and without CABG (Table 1). In individuals with a history of CABG, 96% were using any anti-hypertensive therapy (96% in the empagliflozin group and 98% in the placebo group), 81% were using angiotensin-converting enzyme inhibitor/angiotensin receptor blocker therapy (82% in the empagliflozin group and 80% in the placebo group) and 8% were using mineralocorticoid receptor antagonists (8% in the empagliflozin group and 6% in the placebo group). In addition, 84% of individuals were using statins (83% in the empagliflozin group and 85% in the placebo group), 94% were using antiplatelet/anticoagulant therapy (93% in the empagliflozin group and 95% in the placebo group) and 57% were taking insulin (57% in the empagliflozin group and 56% in the placebo group) (Table 1).

### Cardiovascular outcomes, mortality and renal outcomes

**Fig. 2:**
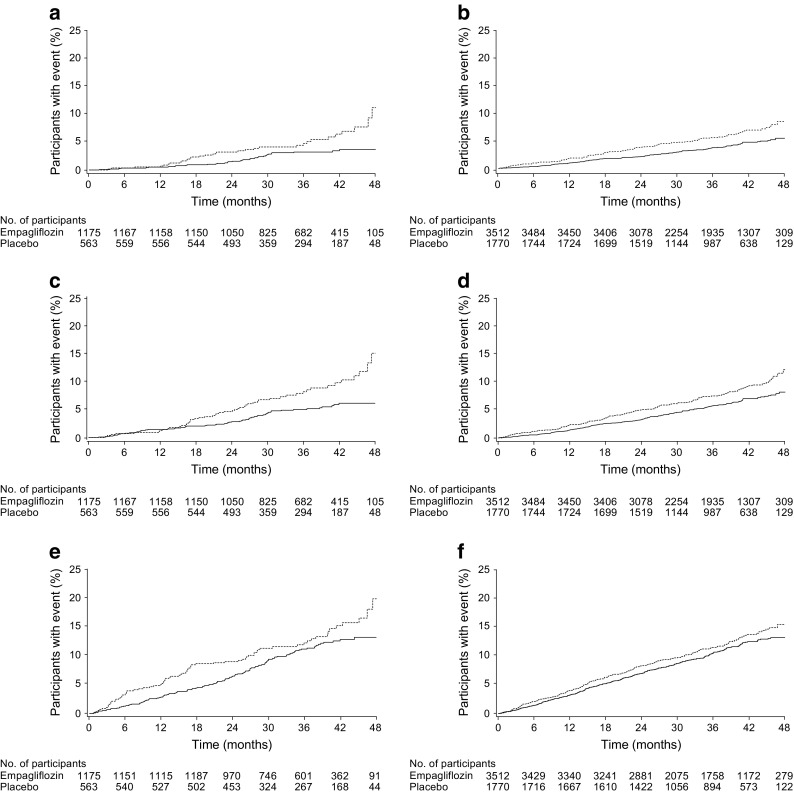
Time to cardiovascular death, all-cause mortality and three-point MACE by history of CABG. (**a**) Cardiovascular death in participants with a history of CABG (HR 0.52 [95% CI 0.32, 0.84]). (**b**) Cardiovascular death in participants without a history of CABG (HR 0.65 [95% CI 0.51, 0.84]). (**c**) All-cause mortality in participants with a history of CABG (HR 0.57 [95% CI 0.39, 0.83]). (**d**) All-cause mortality in participants without a history of CABG (HR 0.73 [95% CI 0.59, 0.90]). (**e**) Three-point MACE in participants with a history of CABG (HR 0.80 [95% CI 0.60, 1.06]). (**f**) Three-point MACE in participants without a history of CABG (HR 0.88 [95% CI 0.74, 1.04]). Kaplan–Meier estimates in participants treated with ≥1 dose of study drug. HR and 95% CI are based on Cox regression analyses. Solid line, empagliflozin; dashed line, placebo. No., number

In participants with a history of CABG at baseline, empagliflozin was associated with a 48% reduction in the risk of cardiovascular death (HR 0.52 [95% CI 0.32, 0.84]) (Figs 1 and 2a). This was consistent with the overall trial population (HR 0.62 [95% CI 0.49, 0.77]), and there was no significant difference between participants with and without a history of CABG at baseline (*p* = 0.3976 for treatment by subgroup interaction) (Fig. 1). Results appeared to be consistent across subgroups by time from CABG to randomisation (ESM Table 1). In participants with a history of CABG at baseline, empagliflozin reduced the risk of all-cause mortality by 43% (HR 0.57 [95% CI 0.39, 0.83]) (*p* = 0.2695 for interaction between subgroups of participants with and without CABG at baseline) (Figs 1 and 2b).

Consistent with the overall trial population, the HR for three-point MACE with empagliflozin vs placebo was 0.80 (95% CI 0.60, 1.06) in participants with a history of CABG at baseline (Figs 1 and 2c). This was consistent with participants without a history of CABG (HR 0.88 [95% CI 0.74, 1.04]) (*p* = 0.5586 for treatment by subgroup interaction). There was no difference between empagliflozin and placebo in the risk of myocardial infarction or stroke in participants with or without a history of CABG (Fig. 1). The HR for four-point MACE with empagliflozin vs placebo was 0.89 (95% CI 0.68, 1.15) in participants with a history of CABG at baseline, and this was consistent with participants without a history of CABG (HR 0.89 [95% CI 0.76, 1.04]) (*p* = 0.9933 for treatment by subgroup interaction). Of participants with a history of CABG at baseline, a smaller proportion treated with empagliflozin (7.7%) than placebo (9.8%) underwent ≥1 coronary revascularisation procedure during the trial. Of participants who underwent coronary revascularisation, most had only one such procedure (ESM Table 2).

**Fig. 3:**
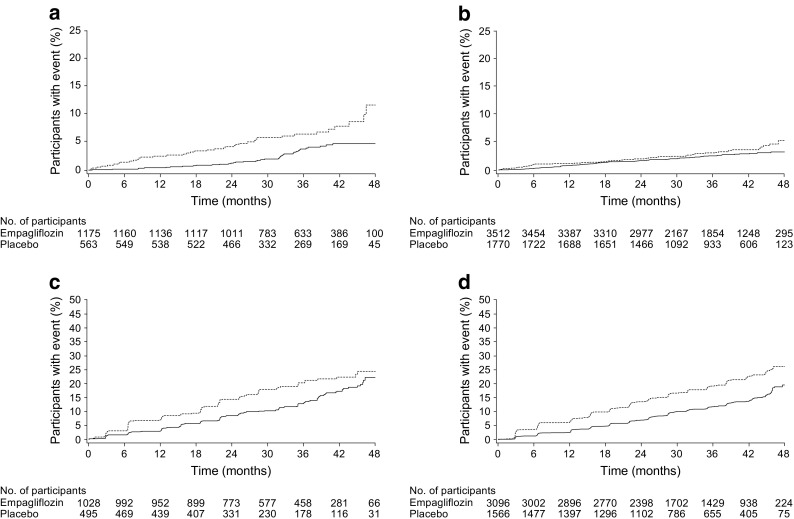
Time to hospitalisation for heart failure and incident or worsening nephropathy by history of CABG. (**a**) Time to hospitalisation for heart failure in participants with a history of CABG (HR 0.50 [95% CI 0.32, 0.77]). (**b**) Time to hospitalisation for heart failure in participants without a history of CABG (HR 0.76 [95% CI 0.54, 1.06]). (**c**) Incident or worsening nephropathy in participants with a history of CABG (HR 0.65 [95% CI 0.50, 0.84]). (**d**) Incident or worsening nephropathy in participants without a history of CABG (HR 0.60 [95% CI 0.51 0.69]). Kaplan–Meier estimates in participants treated with ≥1 dose of study drug, except for incident or worsening nephropathy, which was analysed in participants who received ≥1 dose of study drug who did not have macroalbuminuria at baseline, had serum creatinine measurements at baseline and after baseline and had post-baseline urine albumin-to-creatinine ratio measurements. HR and 95% CI are based on Cox regression analyses. Solid line, empagliflozin; dashed line, placebo. No., number

Consistent with the overall trial population, empagliflozin reduced the risk of hospitalisation for heart failure by 50% in participants with a history of CABG at baseline (HR 0.50 [95% CI 0.32, 0.77]). Risk reductions were consistent between participants with and without a history of CABG (*p* = 0.1359 for treatment by subgroup interaction) (Figs 1 and 3a). Consistent with the overall trial population, the HR for the composite of heart failure hospitalisation or cardiovascular death in participants with a history of CABG was 0.52 (95% CI 0.37, 0.74). Risk reductions were consistent between participants with and without a history of CABG (*p* = 0.1264 for treatment by subgroup interaction) (Fig. 1). Also consistent with the overall trial population, empagliflozin reduced the risk of incident or worsening nephropathy by 35% in participants with a history of CABG at baseline (HR 0.65 [95% CI 0.50, 0.84]). This was consistent between participants with and without a history of CABG (*p* = 0.5673 for interaction) (Figs 1 and 3b).

### Adverse events

Adverse events are summarised in Table 2. Compared with placebo, the proportions of participants with serious adverse events or adverse events leading to discontinuation were lower or similar with empagliflozin in participants with and without a history of CABG. The proportions of participants with confirmed hypoglycaemic adverse events and hypoglycaemic adverse events requiring assistance were greater in participants with a history of CABG, but were similar between empagliflozin and placebo.

**Table 2:** Adverse events

Event	Participants with a history of CABG		Participants without a history of CABG	
	Empagliflozin (*n* = 1175)	Placebo (*n* = 563)	Empagliflozin (*n* = 3512)	Placebo (*n* = 1770)
Adverse event	1092 (92.9)	532 (94.5)	3138 (89.4)	1607 (90.8)
Serious adverse event^a^	490 (41.7)	286 (50.8)	1299 (37.0)	702 (39.7)
Adverse event leading to discontinuation of study drug	215 (18.3)	122 (21.7)	598 (17.0)	331 (18.7)
Confirmed hypoglycaemic adverse event^b^	409 (34.8)	208 (36.9)	894 (25.5)	442 (25.0)
Requiring assistance	23 (2.0)	11 (2.0)	40 (1.1)	25 (1.4)
Adverse event consistent with urinary tract infection^c^	187 (15.9)	78 (13.9)	655 (18.7)	345 (19.5)
Adverse event consistent with genital infection^d^	91 (7.7)	12 (2.1)	210 (6.0)	30 (1.7)
Adverse event consistent with volume depletion^e^	80 (6.8)	36 (6.4)	159 (4.5)	79 (4.5)
Acute renal failure^f^	68 (5.8)	62 (11.0)	178 (5.1)	93 (5.3)
Acute kidney injury	14 (1.2)	21 (3.7)	31 (0.9)	16 (0.9)
Thromboembolic event^f^	9 (0.8)	6 (1.1)	21 (0.6)	14 (0.8)
Bone fracture^g^	49 (4.2)	24 (4.3)	130 (3.7)	67 (3.8)
Lower limb amputation^h^	27 (2.3)	14 (2.5)	61 (1.7)	29 (1.6)

Data are *n* (%) of participants treated with ≥1 dose of study drug in whom ≥1 such adverse event was reported. Participants can be included in ≥1 category. Events that occurred during treatment or ≤7 days after the last dose of study drug are presented

^a^Defined as an adverse event that resulted in death, was immediately life-threatening, resulted in persistent or marked disability/incapacity, required or prolonged participant hospitalisation, was a congenital anomaly/birth defect or was deemed serious for any other reason

^b^Plasma glucose ≤3.39 mmol/l and/or requiring assistance

^c^Based on 79 Medical Dictionary for Regulatory Activities (MedDRA) preferred terms

^d^Based on 88 MedDRA preferred terms

^e^Based on eight MedDRA preferred terms

^f^Based on one standardised MedDRA query

^g^Based on 62 MedDRA preferred terms

^h^Identified from events reported as adverse events, those reported as a ‘medical procedure’ in electronic case report forms or in investigator comments describing adverse events, and via a systematic search of serious adverse event narratives

A greater proportion of participants treated with empagliflozin than placebo had adverse events consistent with genital infections. The proportion of participants with adverse events consistent with volume depletion was greater in participants with a history of CABG, but was similar between empagliflozin and placebo. In participants with a history of CABG, the proportion of participants with acute renal failure (including acute kidney injury) was lower in the empagliflozin group (5.8%) than the placebo group (11.0%) (although statistical tests were not performed). The proportion of participants with lower limb amputations was balanced between the empagliflozin and placebo groups in participants with and without CABG.

## Discussion

In this post hoc subanalysis of data from the EMPA-REG OUTCOME® trial, we report findings in participants with type 2 diabetes and a history of CABG that are consistent with those in the overall trial population [8, 14]. In participants with a history of CABG, cardiovascular death was reduced by 48%, all-cause mortality by 43%, hospitalisation for heart failure by 50% and incident or worsening nephropathy by 35% with empagliflozin vs placebo.

In addition to a greater burden of atherosclerosis, diabetes predisposes individuals to diffuse coronary lesions that are more vulnerable and often not amenable to complete revascularisation [15]. People with diabetes also have specific myocardial defects and are known to exhibit high rates of diastolic dysfunction, placing them at high risk of heart failure [6, 16, 17]. Although much attention has focused on reducing macrovascular events through antiplatelet, antithrombotic and cholesterol-lowering approaches, limited success has been achieved in the prevention of heart failure and mortality following CABG in individuals with type 2 diabetes. Our analyses illuminate the benefits of empagliflozin in individuals with type 2 diabetes and a history of CABG, demonstrating clinically important reductions in important adverse cardiovascular and renal outcomes in this high-risk population when empagliflozin was given in addition to standard of care.

Several mechanisms have been suggested to underlie the beneficial effects of empagliflozin on heart failure and cardiovascular death [18, 19]. As an SGLT2 inhibitor, empagliflozin promotes renal glycosuria and natriuresis [20, 21]. Whereas glycosuria leads to an improvement in glycaemic control, it has been proposed that the ensuing natriuresis and osmotic diuresis reduces preload and ventricular stress [18, 22]. Individuals with type 2 diabetes and cardiovascular disease have a higher body sodium content and, in many instances, are in pre-clinical heart failure or volume overload; thus, recalibration of sodium/volume balance may improve outcomes. In a cohort of ten post-CABG participants with type 2 diabetes, empagliflozin reduced indices of diastolic dysfunction within a 3 month period [23]. In non-clinical models, empagliflozin has been shown to prevent worsening of cardiac failure [24, 25]. Empagliflozin may also improve myocardial energetics through increasing ketone production, yielding ATP at a more efficient rate than other substrates, although this concept remains to be proven [26]. Further, empagliflozin may have SGLT2-independent effects that attenuate myocardial sodium-hydrogen exchange, promoting calcium efflux through cardiomyocytes [27]. The renal benefits of empagliflozin appear to stem from natriuresis-induced tubuloglomerular feedback, which is believed to cause afferent arteriolar vasoconstriction, reducing intraglomerular hypertension [28–30]. Large clinical studies will examine the role of empagliflozin in the treatment of cardiac failure both in participants with reduced and preserved ejection fraction (NCT03057977, NCT03057951) and in the treatment of participants with chronic kidney disease [31].

Guidelines published by the American Heart Association highlight that the majority of evidence for secondary prevention in individuals who have undergone CABG has been derived and/or extrapolated from studies of broader populations of participants with coronary disease [32]. Very few subanalyses of large trials examining subgroups of participants after CABG are available to help guide practice. The only Class IIa recommendation (level of evidence B) that exists for people who have had CABG with respect to diabetes is to strive to achieve an HbA_1c_ of 53 mmol/mol (7%) [32]. There are no specific recommendations on the type of glucose-lowering agents that should be used. Our data provide additional information to help guide clinicians about the choice of glucose-lowering agent for secondary prevention in individuals with type 2 diabetes after CABG. From a practical point of view, surgeons are to be reminded that SGLT2 inhibitors are not indicated in individuals with type 1 diabetes, although trials are ongoing to establish the benefit:risk in this population. Moreover, although hypoglycaemia does not occur with these agents per se, they may increase hypoglycaemia when combined with a sulfonylurea or insulin. Thus, in certain clinical scenarios, a collaborative approach between surgeons and endocrinologists or primary care clinicians will be required. Empagliflozin was not associated with an increased risk of lower limb amputation in participants with or without a history of CABG, similar to observations in the overall trial population [33] and in subgroups by peripheral artery disease at baseline [34].

The EMPA-REG OUTCOME® trial had several strengths. It was a large international trial in which participants received the study drug in addition to standard of care. Cardiovascular events and deaths were adjudicated by blinded committees. Vital status was available for 99% of participants [8]. Participants were identified as having CABG at baseline based directly on case report forms. However, a few limitations to our findings in CABG subgroups must be acknowledged. These analyses were post hoc. Data specific to surgical revascularisation (e.g. number of grafts, use of arterial or venous grafts, on- or off-pump) are not available, nor are biomarker data (e.g. B-type natriuretic peptide). No echocardiograms were performed and baseline indices of left ventricular systolic or diastolic function are unknown. Our analyses were not adjusted for changes in background glucose-lowering therapy or cardiovascular medications.

### Conclusions

In participants with type 2 diabetes and a history of CABG, empagliflozin given in addition to standard of care was associated with significant reductions in cardiovascular and all-cause mortality, hospitalisation for heart failure and incident or worsening nephropathy compared with placebo. The relative risk reductions with empagliflozin were consistent with those observed in the overall EMPA-REG OUTCOME® trial population. Absolute risk reductions, particularly for hospitalisation for heart failure, were numerically greater in participants with a history of CABG, reflecting the higher event rates in these participants (although no statistical tests were performed to compare absolute risk reductions). These data have important implications for secondary prevention pharmacotherapy after CABG in individuals with type 2 diabetes.

## Electronic supplementary material

ESM (PDF 153 kb)

## Data Availability

The datasets generated during and/or analysed during the current study are available from the corresponding author on reasonable request.

## Abbreviations

CABG: Coronary artery bypass graft
eGFR: estimated GFR
FDA: Food and Drug Administration
MACE: Major adverse cardiovascular events
MDRD: Modification of diet in renal disease
PCI: Percutaneous coronary intervention
SGLT2: Sodium–glucose cotransporter 2
