# Supplementary material for: Empagliflozin reduces cardiovascular events, mortality and renal events in participants with type 2 diabetes after coronary artery bypass graft surgery: subanalysis of the EMPA-REG OUTCOME® randomised trial
Source: Diabetologia. 2018 May 19;61(8):1712–23. doi: 10.1007/s00125-018-4644-9 (PMC6061159; doi:10.1007/s00125-018-4644-9)
Supplement: Supplementary file 1 — (PDF 153 kb) [file 125_2018_4644_MOESM1_ESM.pdf]

Empagliflozin reduces cardiovascular events, mortality and renal events in participants with type 2 diabetes after coronary artery bypass graft surgery: subanalysis of the EMPA-REG OUTCOME® randomised trial (Verma S et al).

Electronic Supplementary Material (ESM)

ESM Fig. 1. Study disposition.[1] From N Engl J Med, Zinman B, Wanner C, Lachin JM, Fitchett D, Bluhmki E, Hantel S, Mattheus M, Devins T, Johansen OE, Woerle HJ, Broedl UC, Inzucchi SE, Empagliflozin, Cardiovascular Outcomes, and Mortality in Type 2 Diabetes, Volume No. 373, Page No. 2117–28. Copyright © (2018) Massachusetts Medical Society. Reprinted with permission from Massachusetts Medical Society.

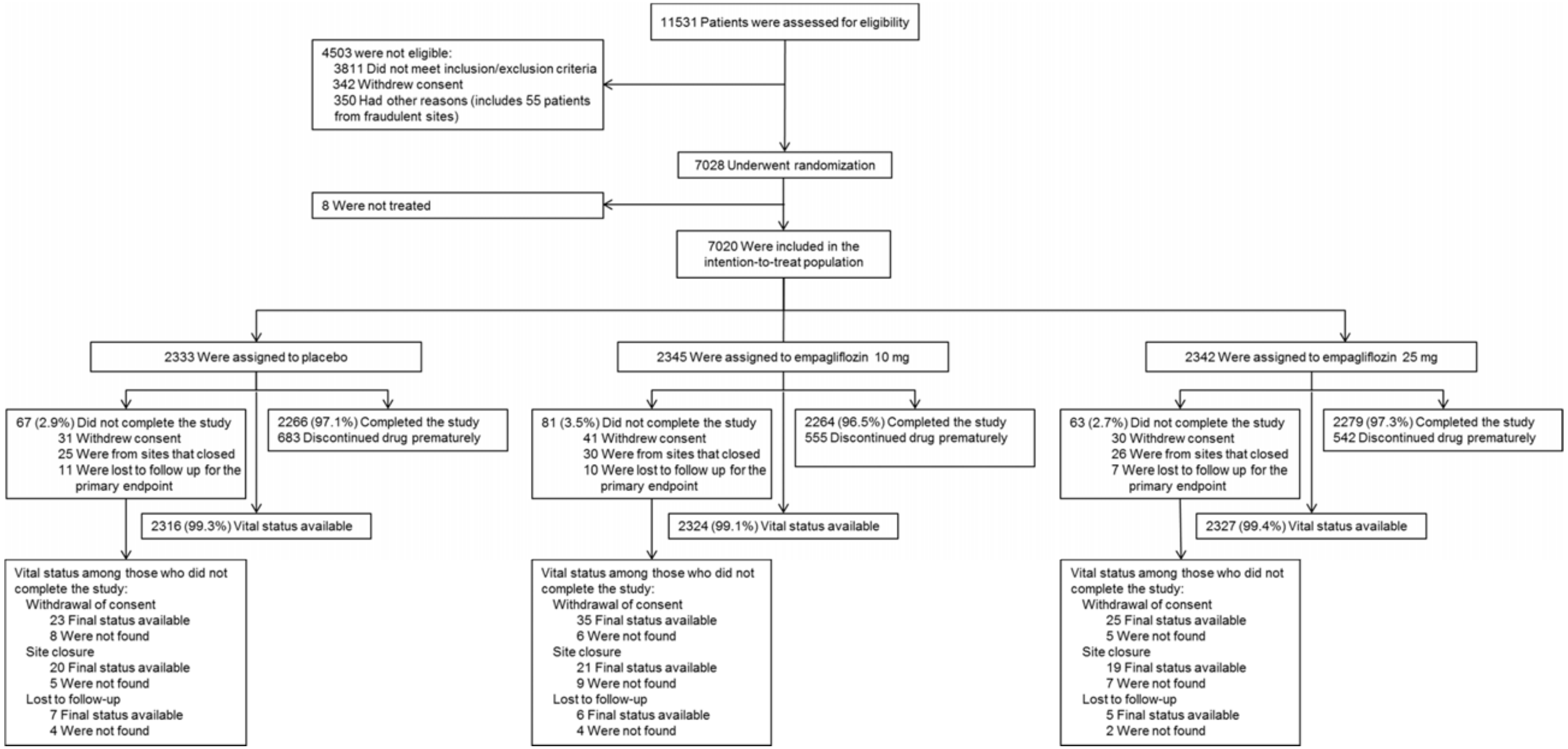

ESM Table 1. Outcomes by time from coronary artery bypass graft (CABG) to randomisation

Outcome	Time from CABG to randomisation	Placebo	Empagliflozin
		n with event/N (%)	n with event/N (%)
**CV death**	≤5 years	9/207 (4.3)	13/472 (2.8)
	>5 to ≤10 years	10/170 (5.9)	10/373 (2.7)
	>10 years	13/181 (7.2)	12/322 (3.7)
**Hospitalisation for heart failure**	≤5 years	9/207 (4.3)	11/472 (2.3)
	>5 to ≤10 years	11/170 (6.5)	9/373 (2.4)
	>10 years	18/181 (9.9)	18/322 (5.6)
**All-cause mortality**	≤5 years	11/207 (5.3)	21/472 (4.4)
	>5 to ≤10 years	22/170 (12.9)	19/373 (5.1)
	>10 years	17/181 (9.4)	19/322 (5.9)

Participants treated with ≥1 dose of study drug.

ESM Table 2. Coronary revascularisation procedures in participants with history of coronary artery bypass graft at baseline

Coronary revascularisation procedures during the trial	Placebo (N=563)	Empagliflozin (N=1175)
Participants with ≥1 procedure	55 (9.8)	91 (7.7)
1 procedure	48 (8.5)	84 (7.1)
2 procedures	7 (1.2)	2 (0.2)
3 procedures	0	4 (0.3)
4 procedures	0	0
5 procedures	0	1 (0.1)

Data are *n (%)* in participants treated with ≥1 dose of study drug.

References in ESM

1. Zinman B, Wanner C, Lachin JM et al. Empagliflozin, cardiovascular outcomes, and mortality in type 2 diabetes. N Engl J Med 2015;373:2117–2128.
